# Preliminary Efficacy, Feasibility, and Perceived Usefulness of a Smartphone-Based Self-Management System With Personalized Goal Setting and Feedback to Increase Step Count Among Workers With High Blood Pressure: Before-and-After Study

**DOI:** 10.2196/43940

**Published:** 2023-07-21

**Authors:** Tomomi Shibuta, Kayo Waki, Kana Miyake, Ayumi Igarashi, Noriko Yamamoto-Mitani, Akiko Sankoda, Yoshinori Takeuchi, Masahiko Sumitani, Toshimasa Yamauchi, Masaomi Nangaku, Kazuhiko Ohe

**Affiliations:** 1 Department of Healthcare Information Management The University of Tokyo Hospital Tokyo Japan; 2 Department of Biomedical Informatics Graduate School of Medicine The University of Tokyo Tokyo Japan; 3 Department of Diabetes and Metabolic Diseases Graduate School of Medicine The University of Tokyo Tokyo Japan; 4 Department of Gerontological Home Care and Long-term Care Nursing Graduate School of Medicine The University of Tokyo Tokyo Japan; 5 Department of Biostatistics, School of Public Health Graduate School of Medicine The University of Tokyo Tokyo Japan; 6 Department of Pain and Palliative Medicine The University of Tokyo Hospital Tokyo Japan; 7 Division of Nephrology and Endocrinology Graduate School of Medicine The University of Tokyo Tokyo Japan

**Keywords:** behavior change, blood pressure, feasibility studies, goal setting, mobile health, mHealth, self-control, self-efficacy, self-regulation, smartphone, step count, walking, workplace, mobile phone

## Abstract

**Background:**

High blood pressure (BP) and physical inactivity are the major risk factors for cardiovascular diseases. Mobile health is expected to support patients’ self-management for improving cardiovascular health; the development of fully automated systems is necessary to minimize the workloads of health care providers.

**Objective:**

The objective of our study was to evaluate the preliminary efficacy, feasibility, and perceived usefulness of an intervention using a novel smartphone-based self-management system (DialBetes Step) in increasing steps per day among workers with high BP.

**Methods:**

On the basis of the Social Cognitive Theory, we developed personalized goal-setting and feedback functions and information delivery functions for increasing step count. Personalized goal setting and feedback consist of 4 components to support users’ self-regulation and enhance their self-efficacy: goal setting for daily steps, positive feedback, action planning, and barrier identification and problem-solving. In the goal-setting component, users set their own step goals weekly in gradual increments based on the system’s suggestion. We added these fully automated functions to an extant system with the function of self-monitoring daily step count, BP, body weight, blood glucose, exercise, and diet. We conducted a single-arm before-and-after study of workers with high BP who were willing to increase their physical activity. After an educational group session, participants used only the self-monitoring function for 2 weeks (baseline) and all functions of DialBetes Step for 24 weeks. We evaluated changes in steps per day, self-reported frequencies of self-regulation and self-management behavior, self-efficacy, and biomedical characteristics (home BP, BMI, visceral fat area, and glucose and lipid parameters) around week 6 (P1) of using the new functions and at the end of the intervention (P2). Participants rated the usefulness of the system using a paper-based questionnaire.

**Results:**

We analyzed 30 participants (n=19, 63% male; mean age 52.9, SD 5.3 years); 1 (3%) participant dropped out of the intervention. The median percentage of step measurement was 97%. Compared with baseline (median 10,084 steps per day), steps per day significantly increased at P1 (median +1493 steps per day; *P*<.001), but the increase attenuated at P2 (median +1056 steps per day; *P*=.04). Frequencies of self-regulation and self-management behavior increased at P1 and P2. Goal-related self-efficacy tended to increase at P2 (median +5%; *P*=.05). Home BP substantially decreased only at P2. Of the other biomedical characteristics, BMI decreased significantly at P1 (*P*<.001) and P2 (*P*=.001), and high-density lipoprotein cholesterol increased significantly only at P1 (*P*<.001). DialBetes Step was rated as useful or moderately useful by 97% (28/29) of the participants.

**Conclusions:**

DialBetes Step intervention might be a feasible and useful way of increasing workers’ step count for a short period and, consequently, improving their BP and BMI; self-efficacy–enhancing techniques of the system should be improved.

## Introduction

### Background

Cardiovascular diseases (CVDs) are the leading cause of death worldwide [1]; they also affect patients’ quality of life and health care expenditures. High blood pressure (BP) is one of the major risk factors for CVDs [2,3]. BP levels have dose-response associations with mortality from CVDs in Japanese cohorts [4]. Aerobic exercise, such as brisk walking, is effective [5] and recommended for lowering BP [6,7]. However, physical inactivity remains as a critical public health issue worldwide [8,9]. In Japan, although the second term of the National Health Promotion Movement in the 21st Century has established goals for steps per day (9000 steps for men and 8500 steps for women who are aged 20-64 years) [10], the mean steps per day has been gradually decreasing since 2000 [11] and falls short of the goals.

Mobile health (mHealth) is expected to increasingly support people’s self-management for improving cardiovascular health, such as physical activity promotion and BP control [12]. mHealth interventions with feedback from care providers were associated with significant reduction in BP [13]. A meta-analysis of mobile phone–based weight loss interventions suggested that personal contact between participants and intervention staff and more frequent interactions were associated with weight reduction [14]. However, interventions needing time investment from care providers or intervention staff may prevent scalability under the limited workforce in the current superaged society. In our previous studies of a smartphone-based self-management system for workers with abdominal obesity [15,16], creating monthly feedback reports including lifestyle advice placed a heavy burden on health care providers. Therefore, the development of mHealth systems with fully automated feedback and individualized advice is necessary to minimize their workloads.

Theory is helpful in guiding hypothesized mechanisms of behavior change during the development and evaluation of interventions [17]. The Social Cognitive Theory (SCT) [18] has been used most frequently as a framework for interventions targeting CVD risk factors [19] because it specifies techniques for changing the core determinant of health behavior, namely self-efficacy [20]. Interventions based on the SCT had significant effect on increasing physical activity in survivors of cancer [21]. However, few mobile apps focusing on physical activity have been developed and evaluated based on the SCT.

### Objective

We developed automated feedback and individualized advice functions to increase step count based on the SCT. We added them to the extant smartphone app (DialBeticsLite) [15,16], which facilitates the recording of physical parameters (BP, body weight, and blood glucose) and health behavior related to CVD prevention (diet, exercise, and daily step count) and provides brief evaluation messages about physical parameters and general diet advice. We focused on daily step count because it is the best-known and objectively measurable key indicator of aerobic physical activity. Longitudinal studies have demonstrated that great daily step counts were associated with low risk of all-cause mortality [22,23] and CVDs [23]. This paper describes the system development and a pilot study of workers with high BP aiming to evaluate the preliminary efficacy, feasibility, and perceived usefulness of an intervention using the new system (DialBetes Step) in increasing step count.

## Methods

### System Development

#### Theoretical Framework

We used the SCT and the *stages of change* in the Transtheoretical Model [24,25] as the theoretical basis of DialBetes Step.

We focused on 3 constructs of the SCT to create new functions to increase step count: *self-efficacy*, *self-regulation*, and *behavioral capability*. *Self-efficacy* is the conviction that one can successfully perform a behavior that leads to an outcome, which can be changed through certain techniques (eg, mastery experience by *performance accomplishments* and *verbal persuasion*) [26]. *Self-regulation* is the process through which an individual observes their own behavior (self-monitoring), judges it by comparison with personal standards (self-set goals), and gives self-evaluative or tangible self-reactions (self-reward) to modify their own behavior [18,27]. Interventions combining self-monitoring of behavior with at least one of the other self-regulation techniques (eg, goal setting and feedback) were more effective in increasing physical activity [28]. Self-regulation techniques partly overlap with techniques that improve self-efficacy [29]. For example, graded goal setting contributes to accumulating mastery experience, and feedback is a means of verbal persuasion [30]. Therefore, we hypothesized that the enhancement of self-regulation would increase physical activity not only directly but also by mediating improvements in self-efficacy [31,32].

Finally, we developed personalized goal-setting and feedback functions, focusing on daily step count to support users’ self-regulation and enhance their self-efficacy. We intended for users to set step goals in gradual increments to have many mastery experiences through goal achievement. We also included information delivery functions to enhance users’ *behavioral capability* (ie, knowledge and skill) [27].

The *stages of change* were used to define the system’s target population. We selected people in the *contemplation* stage (in which they are seriously thinking about overcoming a problem) and the *preparation* stage (in which they intend to change behavior in the very near future or are engaging in small behavior changes) as our target population. Patients in these stages were more likely to be willing to use a self-management tool based on information and communication technology than those in the precontemplation stage [33].

#### Development Process

**Figure 1:**
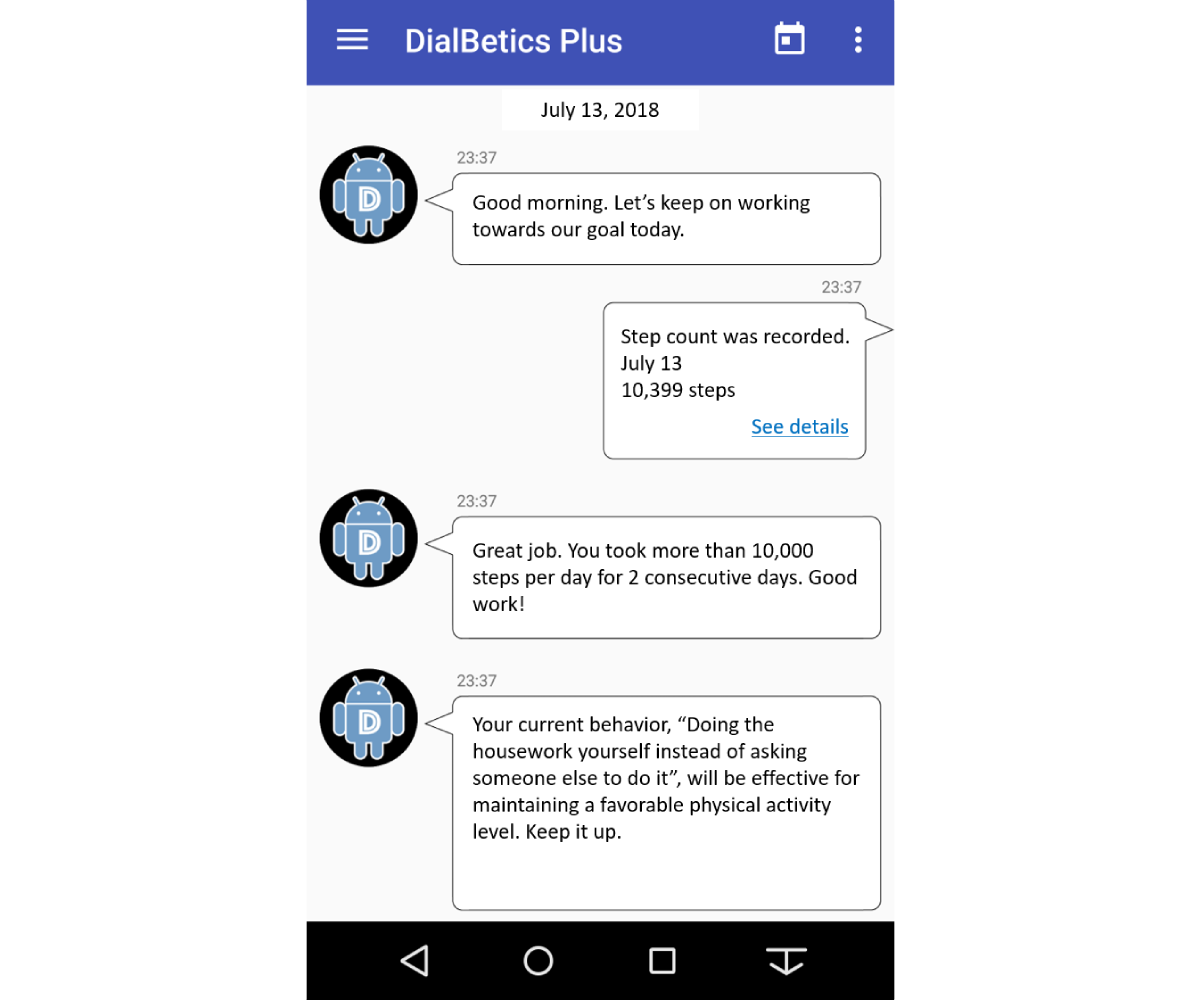
An example of the timeline screenshot of the DialBetes Step app. We copied and transformed the Google material (Android robot) following the Creative Commons 3.0 Attribution license. "DialBetics Plus" was the name of the app when the study was conducted.

We decided on the conceptual framework described previously, content, and algorithms of the new functions by reviewing the literature, including previous information and communication technology–based intervention studies [34-48] on increasing physical activity based on the SCT. We also had continuous discussions within the interdisciplinary team of medical and nursing researchers, such as a registered nurse (TS), endocrinologists (KW and KM), a cardiologist, and a specialist in behavioral medicine. We made an interactive timeline screen on which users could see messages from the system (Figure 1). After the implementation of new functions in DialBeticsLite, the authors (TS, AI, and NYM) and 5 healthy volunteers tested the user interface of the timeline screen and the accuracy of the new function algorithms.

#### The New Functions

The functions for increasing step count in DialBetes Step were divided into self-monitoring, information delivery, and personalized goal setting and feedback. Overall, DialBetes Step included 18 behavior change techniques defined in the Behavior Change Technique Taxonomy version 1 [49] (Multimedia Appendix 1).

DialBeticsLite already has functions to record daily step count and show them as a list and a graph (self-monitoring function). Information delivery functions comprise general information to increase step count, individualized advice to promote physical activity, and individualized information for safe physical activity. When starting the use of the new functions, users can see general information (an action list consisting of 12 actions [50,51]; Textbox S1 in Multimedia Appendix 2) for increasing the step count. We created individualized advice to promote physical activity by modifying the physical activity–related algorithms of Lifestyle Intervention Support Software for Diabetes Prevention [52]. When the user does not meet their step goal, the system recommends one of the behaviors the user “never” or “rarely” does from the items of the “evaluation scale for self-management behavior related to physical activity of type 2 diabetic patients” (ES-SMBPA-2D) [53].

Functions of personalized goal setting and feedback consist of 4 components: goal setting for daily steps, positive feedback, action planning, and barrier identification and problem-solving.

First, DialBetes Step helps users set their own step goals once a week. The process of goal setting involves system-suggested goals, self-setting of goals, and system-suggested goal adjustments according to the user’s self-efficacy in goal achievement. As there were insufficient studies for a way to calculate appropriate step goals automatically, we made algorithms for goal suggestion by simulation using data from our previous study [54]. Therefore, the system-suggested goals are based on the user’s daily step counts at baseline (P0; at week 1) or the number of days in the previous week the user achieved their step goal (at and after week 2) [45,55]. The maximum goal the system suggests and the user can input are both 15,000 steps per day. Users input their confidence in achieving the goal on >4 days of the week, from 0% to 100%. If their confidence level is from 70% to 90%, the system judges the goal as appropriate [41]; otherwise, the system recommends changing the goal. Thus, DialBetes Step allows users to set challenging yet attainable goals that they were likely to achieve with a little more effort [56,57].

Second, DialBetes Step gives users various types of positive feedback to recognize their efforts in increasing their step count: daily feedback about the user’s step goal achievement (Figure 1), feedback about the user’s favorable physical activity behaviors (Figure 1), and weekly feedback. We created feedback about the user’s favorable physical activity behaviors by modifying the algorithms of Lifestyle Intervention Support Software for Diabetes Prevention [52]. When the user meets their step goal, the system displays a message that recognizes one of the behaviors from the ES-SMBPA-2D the user does “often” or “always” as favorable and that encourages them to continue the behavior. For the other types of feedback, we created algorithms with conditional equations to select one of the messages automatically. Weekly feedback includes the mean steps per day, the number of days on which the user achieved the step goal, mean values of physical parameters in the past week, and feedback messages about changes in each parameter.

Third, DialBetes Step helps users make and review an action plan to achieve their step goal once a week after a step goal is determined. Users choose as many actions from the action list (Textbox S1 in Multimedia Appendix 2) as they plan to accomplish or input any other action in a free write-in column.

In addition to action planning, DialBetes Step helps users identify barriers to walking and think of possible solutions. Users rate current or future barriers instead of reviewing their action plan, depending on their mean steps per day and the number of days the user achieved their step goal in the past week. Even if the step goal is reached, it is essential for users to think of possible barriers to walking in the future, to maintain behavior and prevent relapse [58]. We created lists of common current and future barriers to walking from previous studies [59-62] and lists of possible solutions for each barrier (Tables S1 and S2 in Multimedia Appendix 2).

### Design of the Feasibility Study

We conducted a single-arm pilot study of workers at Tokyu Department Store Health Insurance Society in Tokyo, Japan. The primary objective of this study was to evaluate the short-term efficacy, feasibility, and perceived usefulness of DialBetes Step in 6 weeks. We assumed a clinically meaningful change in steps per day to be 1000 steps, as the Japanese physical activity guideline recommends a 10-minute increase in physical activity per day [51], and 100 steps per minute is an estimate for moderate-intensity walking [63]. We hypothesized that the clinically meaningful change would be reached in 6 weeks based on the system’s goal-setting algorithms. We also assessed whether the short-term efficacy continued after 24 weeks of using DialBetes Step.

This study was registered in the University Hospital Medical Information Network Clinical Trial Registry (UMIN000037970).

### Participants

Employees of 4 private enterprises whose systolic BP had been ≥140 mm Hg at a workplace health checkup in the fiscal year 2017 and who were working in the Tokyo metropolitan area were included in this study. All 4 enterprises were in the service industry (eg, department store) and belong to Tokyu Department Store Health Insurance Society. Employees who had been given nationwide lifestyle intervention [16,64] in the fiscal year 2018 were excluded.

A collaborator in the health insurance society sent written recruitment documents to all candidates via workplace mail. Candidates who judged themselves to meet the 3 entry criteria (not walking enough in general, being willing to increase physical activity through walking, and able to engage in moderate physical activity) applied for participation. Recruitment was continued by the collaborator via telephone or email and by an industrial physician (KW) at the employee outpatient clinic until the number of applicants reached the target described in the following section.

We confirmed the applicants’ eligibility for the study based on the results of their health checkups and a baseline questionnaire. Exclusion criteria were the following: systolic BP of ≥180 mm Hg; recent hemoglobin level <10 g/dL; diabetes other than type 2; experience of any hypoglycemic events within the past 3 months; and pregnancy, lactation, or pregnancy plans in the near future. We consulted another industrial physician to judge the applicants’ eligibility if we had difficulty in making a decision.

**Figure 2:**
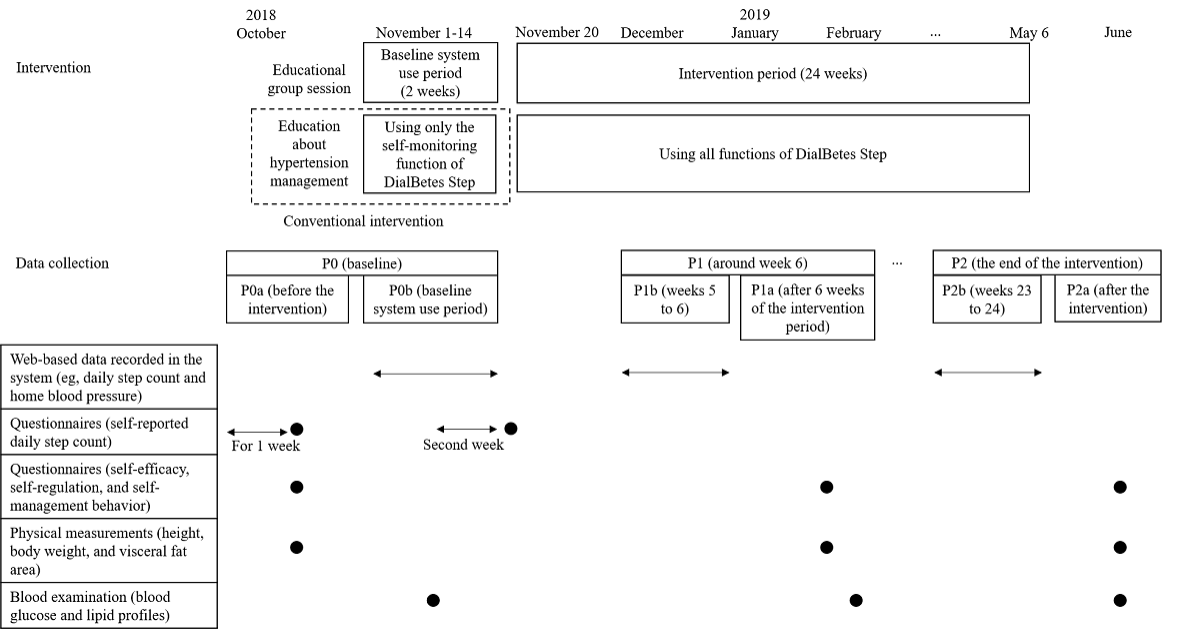
Timeline of the intervention and data collection for outcome evaluation in the before-and-after study among workers with high blood pressure. P0: baseline; P0a: before the intervention; P0b: baseline system use period; P1: around week 6; P1b: weeks 5 to 6; P1a: after 6 weeks of the intervention period; P2: the end of the intervention; P2b: weeks 23 to 24; P2a: after the intervention.

We collected data both via the web (data recorded in DialBetes Step) and offline (paper-based questionnaires, physical measurements, and blood examinations; Figure 2). Participants attended group sessions for offline data collection at the University of Tokyo Hospital at P0, after 6 weeks of the intervention period (P1a), and after the intervention (P2a). Questionnaires at P1a and P2a were sent via email and collected at each group session. Blood samples were collected for examination at the outpatient clinic of the health insurance society at P0 and P1a and during the group session at P2a.

As baseline characteristics, we obtained sex and the date of birth from the health insurance society. The baseline questionnaire before the intervention (P0a) included questions about the participant’s stages of change in increasing step count [65]; outpatient visits for hypertension and diabetes; and routine measurement of home BP, body weight, and step count. Office BP was measured using an automated sphygmomanometer (Omron; HEM-7271T).

After the completion of the study, we compensated the participants with a paper-based report on the results of physical measurements and blood examinations with comments from a registered nurse (TS).

### Ethics Approval

This study was approved by the research ethics committee of the Graduate School of Medicine, the University of Tokyo—approval 11902-(2). We obtained informed consent through 2 processes. Participants initially provided written informed consent to data collection for the short-term evaluation in 6 weeks. At the P2a group session, we informed participants about the remaining data collection through a document and verbal explanation; participants could opt out at their own discretion. We deidentified all study data to protect the participants’ privacy and confidentiality.

### Interventions

#### Educational Group Session (Conventional Intervention)

All participants attended a 2-hour group session in October 2018. The group session included the initial informed consent, baseline data collection, a 40-minute lecture on hypertension management, and a 60-minute lecture with a practice session on how to use DialBetes Step based on a user manual.

#### Use of DialBetes Step

All participants used the system for 26 weeks from November 2018. The study team loaned participants a device set to use the system: a smartphone (Fujitsu; F-02H) in which necessary apps were installed, a home sphygmomanometer (Omron; HEM-7271T), a weight and body composition scale (Omron; HBF-255T), a glucometer (Terumo; MS-FR201B), and a triaxial accelerometer (Terumo; MT-KT02DZ).

For the first 2 weeks, all participants used only the self-monitoring function of DialBetes Step (conventional intervention) for baseline data collection (baseline system use period [P0b]). They measured BP twice a day (before breakfast [morning] and before bedtime [night]) and body weight once a day (morning). Participants whose hemoglobin A_1c_ (glycated hemoglobin) level was ≥6% or fasting blood glucose was ≥100 mg/dL at the latest health checkup also measured blood glucose levels once or twice a day (Multimedia Appendix 3). They recorded the data into the system through Bluetooth communication using a free app (Omron; OMRON connect) or near field communication. All participants wore the accelerometer in a clothes pocket or bag during all waking hours, except when swimming or taking a shower. Participants sent daily step count and calories burned by activity to the system through near field communication every night. Participants also input information about every meal and exercise not recorded by the accelerometer. We asked participants to live their daily life as usual (ie, not to increase physical activity intentionally) during this period.

After the 2-week P0b and a 5-day interval, all participants began using all functions of DialBetes Step for 24 weeks (intervention period). They received the same evaluation messages about physical parameters and diet advice as in the original DialBeticsLite [15,16] and used the new functions.

Participants contacted the study team via telephone or email when they had any trouble with using the system. We monitored the recorded data of the participants through the server every weekday. If a participant did not record accelerometer data for 3 consecutive days, the first author emailed them a reminder.

### Process Evaluation

We calculated percentages for the measurement and recording of each parameter in DialBetes Step during P0b and intervention period. The percentages were calculated by dividing the number of days data were measured and recorded by the number of days in each period. We excluded days without data owing to recording failures, for which participants were not responsible, from the denominator.

We summarized the number of contacts between participants and the study team in each period, including email reminders to record accelerometer data and problems with system use. We categorized problems according to the functions of DialBetes Step.

Participants completed a paper-based questionnaire to evaluate the system during the P1a group session. Participants rated the usefulness and user-friendliness of the overall system and each function on a 4-point Likert scale (good, somewhat good, somewhat bad, or bad). They also responded to modified items about the overall system evaluation [54].

### Outcome Evaluation

#### Primary Outcome

The primary outcome was the change in the mean steps per day recorded in DialBetes Step between P0b and weeks 5 to 6 (P1b) of the intervention period. We also assessed the long-term change between P0b and weeks 23 to 24 (P2b). We excluded data from days on which the participant did not wear the accelerometer according to their self-report or had daily step count <100 steps [66].

To assess the change in the mean steps per day presumably caused by the conventional intervention, participants also recorded daily step count for a week before the educational group session (ie, P0a) and during the second week of P0b on questionnaires if they had measured it using their own devices (eg, smartphones). The P0b questionnaire was sent to participants and returned via email.

#### Secondary Outcomes

Secondary outcomes included changes in another physical activity measure, biomedical outcomes, and antecedent factors of physical activity. We assessed short-term changes between P0 and around week 6 (P1: P1b for web-based data or P1a for offline data) and long-term changes between P0 and the end of the intervention (P2: P2b for web-based data or P2a for offline data) in each outcome. Detailed information about measuring instruments and scales is summarized in Multimedia Appendix 4.

We assessed the changes in the mean calories burned by activity per day (measured using the triaxial accelerometer [67]) and the mean home BP (for morning and night) using the web-based data recorded in DialBetes Step.

Other biomedical outcomes included changes in body weight, BMI, and visceral fat area (VFA) measured in the morning during each group session. Height and body weight were measured using an automatic height-and-weight scale (A&D; AD-6228AP), which subtracted 0.5 kg as the tare from the body weight. The first author or a clinical laboratory technician measured participants’ VFA using DUALSCAN (Omron Colin; HDS-2000), which uses the dual impedance method [68,69].

Blood samples collected in the morning were sent to and examined by LSI Medience Corporation, Tokyo, Japan. We evaluated changes in the levels of hemoglobin A_1c_, fasting blood glucose, triglycerides, and high-density lipoprotein (HDL) cholesterol. We used non-HDL cholesterol as an indicator of hypercholesterolemia instead of low-density lipoprotein cholesterol calculated using the Friedewald formula, as some participants had a triglyceride level of >400 mg/dL at P2a.

We assessed the changes in antecedent factors of physical activity using the questionnaire at P0a, P1a, and P2a, including 2 types of self-efficacy, self-regulation, and self-management behavior related to physical activity. First, we assessed self-efficacy in achieving goals for daily steps (goal-related self-efficacy [70]). On the basis of the scales of self-efficacy for physical activity [71-73], we set 4 levels of incremental step goals (6000, 8000, 10,000, and 12,000 steps per day). Participants rated their degree of confidence in achieving each goal by writing a percentage from 0% to 100%. The score was calculated as the average of the confidence estimates for each goal. Second, we assessed self-efficacy in walking behavior [74]. Participants rated their degree of confidence in engaging in regular walking during 4 difficult situations (when tired, when in a bad mood, when feeling as if they did not have the time, and when it was raining or snowing) on a 5-point Likert scale. Self-regulation was assessed using the Japanese version of the 12-item Physical Activity Self-Regulation scale [75,76]. The scale consists of 6 factors (eg, self-monitoring, goal setting, reinforcements, time management, and relapse prevention), with 2 items each, rated on a 5-point Likert scale. Self-management behavior was assessed using the ES-SMBPA-2D, which consists of 9 factors, with a total of 32 items rated on a 5-point Likert scale [53].

### Adverse Events

Adverse events were assessed using questionnaire. First, as DialBetes Step encouraged participants to increase physical activity, we assessed changes in body pain, using 4 items categorized in the body pain cluster in the 25-question Geriatric Locomotive Function Scale [77]. Participants rated those items on a 5-point Likert scale at P0a, P1a, and P2a. Second, we asked participants whether they experienced any changes in their condition or subjective symptoms during system use in the P1a and P2a questionnaires.

### Population for Analyses and Sample Size

We included all participants in the assessment of problems with system use and adverse events. In contrast, we defined the suitable population for the other evaluation (eg, outcome evaluation) as the theoretical target population of DialBetes Step (ie, the contemplation and the preparation stages in the Transtheoretical Model). First, we excluded participants categorized in the precontemplation stage using the P0a questionnaire. Second, we excluded participants who were already taking >15,000 steps per day (equal to the maximum step goal users can set) during P0b because the system encouraged them to maintain that step count rather than increase it.

To calculate the sample size for outcome evaluation, we used the aforementioned clinically meaningful change in steps per day (1000 steps [51,63]) and estimated the SD to be 1800 based on our previous study of DialBeticsLite in Tokyu Department Store Health Insurance Society (K Waki, unpublished data, September 2017). We estimated the necessary sample size to be 28 to detect 0.55 SD of change, with 80% power at the 5% significance level, using G*Power 3.1.9.2 (Heinrich-Heine-Universität) [78]. Anticipating the dropout and ineligibility of a certain number of participants, we set a recruitment target of at least 35 participants.

### Statistical Analyses

Changes in outcomes between P0 and P1 or P2 were tested using the Wilcoxon signed rank test owing to the small sample size and the skewed distribution of variables. We calculated distribution-free 95% CIs of the median using the *ciquantdf* option of the Base SAS 9.3 univariate procedure [79]. As we conducted 2 tests (short-term and long-term analyses) for each outcome, we set the significance level using the 2-tailed test at .025 based on the Bonferroni method.

We excluded participants who did not complete data collection and those without valid data (eg, having breakfast before the physical measurement) from analyses of each outcome. If responses to items of the ES-SMBPA-2D were missing, we substituted the participant’s mean item score in each factor at the same time point for the missing values. If participants reported the start of new medications or increase in dose of any medication for hypertension or dyslipidemia in the P1a or P2a questionnaires, they were excluded from analyses of home BP or lipids, respectively.

We conducted post hoc subgroup analyses to assess whether the changes in steps per day were modified by baseline steps. We divided the participants into 2 groups using the median of the mean steps per day during P0b (<10,000 and >10,000 steps per day) and tested the step changes in each group.

We used SAS Studio 3.8 (SAS Institute) for all analyses.

## Results

### Participant Flow and Baseline Data

**Figure 3:**
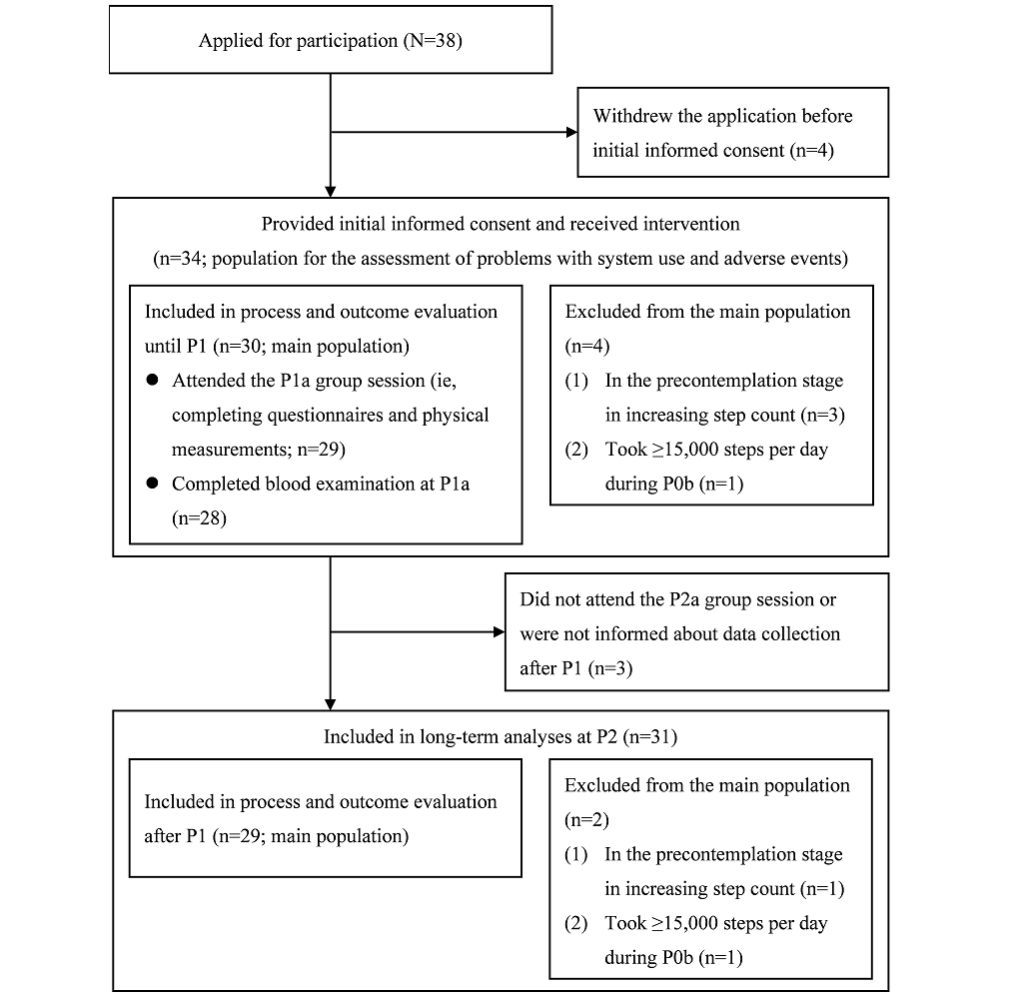
Flow diagram of participants in the before-and-after study. P1: around week 6; P1a: after 6 weeks of the intervention period; P0b: baseline system use period; P2a: after the intervention; P2: the end of the intervention.

Figure 3 shows a flow diagram of participants. Although we could not determine the actual number of candidates who had been sent recruitment documents, 13.2% (34/257) or 13.1% (34/259) of them provided the initial informed consent. All participants were considered to be eligible for inclusion in the study. We included 88% (30/34) participants who met the qualifications for our theoretical target population in the process and outcome evaluation (the main population for analyses). A participant who had plans to be hospitalized for the treatment of arrhythmia at week 5 of the intervention period was excluded from the short-term analyses of the web-based data. We could not inform 9% (3/34) of the participants about data collection after P1; a total of 31 participants (n=29, 94% in the main population) were included in the long-term analyses.

Table 1 shows the baseline characteristics of participants in the main population. Most participants (21/30, 70%) were not overweight or obese.

**Table 1:** Baseline characteristics of the main population for analyses in the before-and-after study among workers with high BP^a^ (n=30).

Characteristics		Values
Sex (male), n (%)		19 (63)
Age (years), mean (SD)		52.9 (5.3)
Systolic BP at hospital (mm Hg), median (IQR)		146 (137-155.5)
Diastolic BP at hospital (mm Hg), median (IQR)		96 (91.5-105)
**BMI (kg/m^2^), median (IQR)**		23.5 (20.9-25.1)
	≥25 (overweight or obesity), n (%)	9 (30)
	<25, n (%)	21 (70)
**Visceral fat area (cm^2^), median (IQR)**		56 (42-88)
	≥100 (abdominal obesity), n (%)	5 (17)
	<100, n (%)	25 (83)
**Stages of change in increasing step count, n (%)**		
	Contemplation	5 (17)
	Preparation	17 (57)
	Action	3 (10)
	Maintenance	5 (17)
Regular outpatient visits for hypertension (yes), n (%)		8 (27)
Regular outpatient visits for type 2 diabetes (yes), n (%)		1 (3)
Routine measurement of home BP (yes), n (%)		13 (43)
Routine measurement of body weight (yes), n (%)		19 (63)
Routine measurement of step count (yes), n (%)		11 (37)

^a^BP: blood pressure.

### Process Evaluation

The median percentages for daily step count measurement were 96.4% (IQR 85.7%-100%) during P0b and 97% (IQR 88.7%-100%) during the intervention period. The median measurement and recording percentages for the other parameters were >85% throughout the study (Table S1 in Multimedia Appendix 5). Only 23% (7/30) of the participants recorded any exercise during the intervention period.

During the study, we sent a total of 37 email reminders to 40% (12/30) of the participants. In total, 79% (27/34) of the participants had a total of 62 problems with system use (Table S2 in Multimedia Appendix 5). Half (31/62, 50%) of them were related to measurement and recording of daily step count (eg, recording failure through near field communication); however, there was no inquiry about the other functions designed to increase step count.

Most participants rated the overall system as useful (16/29, 55%) or somewhat useful (12/29, 41%) for self-management (Table S3 in Multimedia Appendix 5). The function that most participants rated as useful in increasing step count was measurement and recording (25/29, 86%), followed by goal setting (15/29, 52%), weekly feedback (14/29, 48%), and educational group session (11/29, 38%). In contrast, >20% of participants rated the user-friendliness of measurement and recording of daily step count (6/28, 21%), action planning (6/28, 21%), and barrier identification and problem-solving (7/28, 25%) as somewhat bad or bad. Similarly, 76% (22/29) of the participants rated the overall system interface as easy to use (Table S4 in Multimedia Appendix 5). Participants spent an average of 16.5 (SD 11.2; range 3-60) minutes per day in using the system; 90% (26/29) of them reported that the system was worth the time they spent.

### Outcome Evaluation

#### Primary Outcome

The median of the mean steps per day during P0b was >10,000 steps (Tables 2 and 3). They significantly increased at P1b compared with P0b (median +1493 steps per day; *P*<.001). However, the increase attenuated and did not remain significant under the significance level of this study at P2b (median +1056 steps per day; *P*=.04). Week 6 of the intervention period was during the end of the year, and P2b contained many Japanese national holidays, which may have changed participants’ daily activities during those periods. Steps per day significantly increased when we compared P0b and week 5 only (median +1497 steps per day; *P*=.003) or weeks 21 to 22 (median +1005 steps per day; *P*=.008; Multimedia Appendix 6).

Owing to the high number of baseline steps, we conducted post hoc subgroup analyses to assess whether the increases in steps per day were significant in less-active participants, who were more consistent with the theoretical target population of DialBetes Step (Tables S5 and S6 in Multimedia Appendix 5). Steps per day increased at P1b significantly only in the less-active group (median +1451; 95% CI +111 to +3220 steps per day; *P*=.005 vs median +1958; 95% CI −544 to +3055 steps per day; *P*=.05 in the more active group). Both groups did not significantly change steps per day at P2b.

Only 30% (9/30) of the participants were included in the comparison of steps per day between P0a and P0b. The median was 9578 (IQR 8433-10,326) steps per day at P0a and did not change until P0b (median +53 steps per day; *P*=.82).

**Table 2:** Short-term changes from P0^a^ to P1^b^ in outcome variables in the before-and-after study among workers with high BP^c^ (n=30).

Variables		P0a^d^, median (IQR)	P0b^e^, median (IQR)	Changes from P0 to P1	
				Median (95% CI)	*P* value^f^
**Physical activity**					
	Mean steps per day^g^	N/A^h^	10,020 (8548-11,519)	+1493 (+131 to +2584)	<.001
	Mean calories burned by activity (kcal per day)^g^	N/A	265 (248-331)	+50 (+6 to +83)	<.001
**Mean home BP (mm Hg)^g^**					
	Morning—systolic	N/A	131.1 (126.3-141.4)	−0.2 (−3.4 to +2.4)	.66
	Morning—diastolic	N/A	89.7 (83.2-95.3)	−0.6 (−2.6 to +1.8)	.30
	Night—systolic^i^	N/A	126.1 (119.1-136.4)	−3.1 (−6.2 to +2.7)	.23
	Night—diastolic^i^	N/A	80.3 (77.3-84.5)	−1.2 (−5.0 to +1.1)	.44
**Results of physical measurement^j^**					
	Body weight (kg)	66 (57.7-72.4)	N/A	−1.2 (−1.9 to −0.4)	<.001
	BMI (kg/m^2^)	23.7 (21.1-25.1)	N/A	−0.4 (−0.7 to −0.1)	<.001
	Visceral fat area (cm^2^)^k^	56.5 (46-88)	N/A	−1 (−8 to +3)	.18
**Results of blood examination^l^**					
	Hemoglobin A_1c_ (%)	N/A	5.4 (5.1-5.5)	0 (0 to +0.1)	.94
	Fasting blood glucose (mg/dL)	N/A	88 (82-92)	+0.5 (−1 to +2)	.38
	Triglycerides (mg/dL)	N/A	104 (52-149)	−10 (−29 to +9)	.27
	HDL^m^ cholesterol (mg/dL)	N/A	63 (48.5-79)	+3.5 (+1 to +8)	<.001
	Non-HDL cholesterol (mg/dL)	N/A	157.5 (129.5-189)	−3.5 (−18 to +6)	.20

^a^P0: baseline.

^b^P1: around week 6.

^c^BP: blood pressure.

^d^P0a: before the intervention.

^e^P0b: baseline system use period.

^f^Analyzed using Wilcoxon signed rank test.

^g^A participant who was hospitalized during week 5 was excluded (n=29).

^h^N/A: not applicable.

^i^A participant without measured data at P0b was excluded (n=28).

^j^Overall, 10% (3/30) of the participants were excluded—3% (1/30) did not take physical measurements, and the other 7% (2/30) had breakfast on the day of the measurement at P1 (n=27).

^k^An additional participant was excluded because of inability to use the abdominal belt owing to pain (n=26).

^l^Overall 7% (2/30) of the participants who did not take blood tests at P1 were excluded (n=28).

^m^HDL: high-density lipoprotein.

**Table 3:** Long-term changes from P0^a^ to P2^b^ in outcome variables in the before-and-after study among workers with high BP^c^ (n=29).

Variables		P0a^d^, median (IQR)	P0b^e^, median (IQR)	Changes from P0 to P2	
				Median (95% CI)	*P* value^f^
**Physical activity**					
	Mean steps per day^g^	N/A^h^	10,002 (8486-11,908)	+1056 (−651 to +2286)	.04
	Mean calories burned by activity (kcal per day)^i^	N/A	264 (243-331)	+26 (−21 to +69)	.07
**Mean home BP (mm Hg)^j^**					
	Morning—systolic^k^	N/A	132.3 (126.3-141.4)	−3.3 (−9.8 to +2)	.07
	Morning—diastolic^k^	N/A	90.3 (82.3-95.3)	−1.6 (−6.2 to +0.1)	.02
	Night—systolic^k,l^	N/A	125.5 (117-136)	−4.4 (−8.9 to +0.6)	.02
	Night—diastolic^k,l^	N/A	79.6 (76.2-84.3)	−2.4 (−5.8 to −0.9)	<.001
**Results of physical measurement^m^**					
	Body weight (kg)	66.8 (57.6-73.1)	N/A	−1.4 (−2.7 to −0.2)	.001
	BMI (kg/m^2^)	23.7 (21.1-25.2)	N/A	−0.5 (−1.2 to −0.1)	.001
	Visceral fat area (cm^2^)	56.5 (45-89)	N/A	−0.5 (−7 to +7)	.60
**Results of blood examination^m^**					
	Hemoglobin A_1c_ (%)	N/A	5.4 (5.15-5.6)	+0.1 (0 to +0.1)	.22
	Fasting blood glucose (mg/dL)	N/A	88 (84-92.5)	+2.5 (−1 to +6)	.16
	Triglycerides (mg/dL)^n^	N/A	105 (59-144)	−13 (−30 to +5)	.50
	HDL^o^ cholesterol (mg/dL)^n^	N/A	63 (49-73)	+3 (0 to +7)	.23
	Non-HDL cholesterol (mg/dL)^n^	N/A	158 (130-186)	−6 (−22 to +5)	.25

^a^P0: baseline.

^b^P2: the end of the intervention.

^c^BP: blood pressure.

^d^P0a: before the intervention.

^e^P0b: baseline system use period.

^f^Analyzed using Wilcoxon signed rank test.

^g^A participant without measured data at P2 owing to accelerometer trouble was excluded (n=28).

^h^N/A: not applicable.

^i^Overall, 7% (2/29) of the participants without measured or valid data at P2 owing to accelerometer trouble were excluded (n=27).

^j^A participant who intensified their antihypertensive treatment after P1 was excluded (n=28).

^k^Overall, 7% (2/28) and 4% (1/28) of the participants without measured data at P2 for morning and night, respectively, were excluded (n=26 for morning, n=27 for night).

^l^A participant without measured data at P0b was excluded (n=26).

^m^A participant was excluded because he had breakfast on the day of the measurement and blood tests at P2 (n=28).

^n^An additional participant who started taking a cholesterol medication after P1 was excluded (n=27).

^o^HDL: high-density lipoprotein.

#### Secondary Outcomes

As is the case with steps per day, calories burned by activity per day significantly increased only at P1b compared with P0b (Tables 2 and 3). Home BP did not change significantly between P0b and P1b (all *P*>.025); however, it decreased significantly at P2b (all *P*<.025), except for systolic BP in the morning (*P*=.07). Overall, 7% (2/29) of the participants reported decrease in the dose of antihypertensive medications during system use. Body weight and BMI decreased significantly at both P1a (*P*<.001) and P2a (*P*=.001) compared with that at P0a; however, VFA did not change significantly over time (both *P*>.025). The median percentage decrease in body weight was 1.8% at P1a and 2.1% at P2a. For metabolic outcomes, HDL cholesterol increased significantly at P1a compared with that at P0b (*P*<.001); however, the change from P0b to P2a was not significant (*P*=.23).

The median score of goal-related self-efficacy was 72.5% at P0a (Table 4); the baseline score of 21% (6/29) of the participants was 100%. Although it did not change between P0a and P1a, it tended to increase at P2a compared with that at P0a (*P*=.05), but this was not statistically significant. Self-efficacy in walking behavior did not change over time. The total score of self-regulation and scores for self-monitoring, goal setting, reinforcements, and time management significantly increased at both P1a and P2a (all *P*<.025); the score for relapse prevention significantly increased only at P2a (*P*=.02). Scores for most of the factors of self-management behavior significantly increased at both P1a and P2a, except for selecting a suitable place or time for physical activities and creating situations to enhance active behavior. Items in “exercising to stimulate the enjoyment of eating” are not recommended behaviors; however, the corresponding score significantly increased at P1a (*P*=.005).

**Table 4:** Changes in antecedent factors of physical activity in the before-and-after study among workers with high blood pressure (n=29).^a^

Variables		P0a^b^, median (IQR)	Changes from P0a to P1a^c^			Changes from P0a to P2a^d^	
			Median (95% CI)	*P* value^e^	Median (95% CI)		*P* value^e^
**Self-efficacy**							
	Goal-related self-efficacy (%)^f^	72.5 (63.8-92.5)	0 (−7.5 to +12.5)	.38	+5 (−5 to +15)		.05
	Self-efficacy in walking behavior^g^	11 (8-16)	0 (−2 to +2)	.68	0 (−2 to +3)		.48
**Self-regulation^h^**							
	Total score	20 (16-25)	+5 (+2 to +10)	<.001	+4 (+3 to +13)		<.001
	Self-monitoring	4 (2-5)	+2 (+1 to +3)	<.001	+1 (+1 to +2)		<.001
	Goal setting	4 (2-4)	+1 (0 to +2)	.002	+2 (+1 to +3)		<.001
	Eliciting social support	2 (2-2)	0 (0 to 0)	.09	0 (0 to 0)		.14
	Reinforcements	5 (4-6)	+1 (0 to +2)	.005	+1 (+1 to +2)		<.001
	Time management	4 (2-5)	+1 (0 to +2)	<.001	0 (0 to +2)		.007
	Relapse prevention	2 (2-4)	0 (0 to +2)	.11	0 (0 to +2)		.02
**Self-management behavior related to physical activity (range)^i^**							
	Shopping (0-16)	1 (0-5)	+2 (+1 to +4)	<.001	+2 (0 to +4)		<.001
	Household activities (0-16)	4 (0-6)	+1 (0 to +3)	.006	+1 (0 to +4)		<.001
	Exertion (0-16)	7 (5-9)	+2 (0 to +3)	.002	+1 (+1 to +3)		.004
	Commuting activities (0-16)	6 (4-8)	+2 (+1 to +4)	<.001	+2 (+1 to +4)		<.001
	Suitable place or time (0-20)	8 (6-10)	+1 (0 to +3)	.18	+1 (−1 to +4)		.05
	Self-monitoring (0-12)	0 (0-5)	+7 (+5 to +10)	<.001	+4 (+2 to +7)		<.001
	Making a habit (0-12)	2 (0-5)	+2 (+1 to +4)	.001	+1 (0 to +2)		.007
	Exercising for eating (0-8)	1 (0-3)	0 (0 to +1)	.005	0 (0 to +1)		.18
	Creating situation (0-12)	2 (0-4)	+1 (0 to +2)	.07	+1 (0 to +3)		.002

^a^A participant who did not respond to the questionnaire at P1a was excluded (n=29).

^b^P0a: before the intervention.

^c^P1a: after 6 weeks of the intervention period.

^d^P2a: after the intervention.

^e^Analyzed using Wilcoxon signed rank test.

^f^Average of the self-efficacy in achieving 4 incremental step goals (6000; 8000; 10,000; and 12,000 steps per day; range 0-100). A participant with an invalid response at P0a was excluded (n=28).

^g^Measured using the self-efficacy scale for walking behavior (range 4-20). High score indicates high self-efficacy.

^h^Measured using the Japanese version of the 12-item Physical Activity Self-Regulation scale (range 2-10 for each factor and range 12-60 for the total score). High score indicates high frequency of self-regulation.

^i^Measured using the evaluation scale for self-management behavior related to physical activity of type 2 diabetic patients. High score indicates high frequency of self-management behavior.

### Adverse Events

The score of the body pain cluster did not change over time (Table S7 in Multimedia Appendix 5). The item score of lower limb pain (Table S8 in Multimedia Appendix 5) increased by at least one point in 21% (7/33) of the participants at P1a and 19% (6/31) of the participants at P2a. A participant’s item score was 3 points (“considerable pain”) at P1a and P2a (+1 point from P0a). She took >15,000 steps per day during P0b and further increased the steps per day at P1b.

A participant reported daytime sleepiness (at P1a and P2a) and dizziness upon standing (at P1a) as negative changes in their condition during system use. He attributed these symptoms to his short sleeping hours. His home BP did not change between P0b and P1b. In contrast, 15% (5/33) and 16% (5/31) of the participants at each time point, respectively, reported positive changes, such as their body feeling light.

## Discussion

### Principal Findings

We evaluated the preliminary efficacy, feasibility, and perceived usefulness of the intervention using DialBetes Step, a novel smartphone-based self-management system with personalized goal-setting and feedback functions and information delivery functions designed to increase step count among workers with high BP. Participants rarely missed the measurement and recording of data, and there was a low level of attrition. Despite frequent problems with measurement and recording, the system’s interface was rated as easier to use than that of the original DialBeticsLite [15]. Participants increased their mean steps per day along with engaging more frequently in self-regulation and self-management behaviors related to physical activity. Although the increase in steps per day attenuated at P2b, BP and body weight decreased by the end of the study. Intervention using DialBetes Step may be useful in increasing the step count for a short period and improving cardiovascular risk.

After using the new functions in DialBetes Step, most participants increased their step count by >1000 steps per day, an expected clinically meaningful change [51,63] at both P1b and P2b. Previous reviews suggested significant effects of activity trackers on step count and body weight, with moderate certainty of evidence [80]. The median increases in this study were less than those measured in behavior modification interventions using pedometers (approximately 2000-2500 steps per day on average) [63]. Previous studies showed negative correlation between the increase in steps per day and baseline steps [81,82]. Participants in our study were considered to be physically active [63] at P0, and the post hoc subgroup analyses supported these previous findings. Thus, their potential to increase physical activity may have been lower than that of sedentary people. Similarly, physical activity and daily step count have seasonal variation, in that they tend to decrease in winter [83]. Therefore, the increase from P0b (early November) to P1b (late December) in this study is likely to be more meaningful than the observed value.

Home BP decreased significantly only at P2b. BP is also subject to seasonal variation, as it is highest in winter (middle to late January in Japan) and lowest in summer [84]; the observed changes in home BP in P1b might be underestimated. Of the other biomedical outcomes, body weight, BMI, and HDL cholesterol level improved significantly, even though the median values at P0 were within the normal range. These improvements were almost the same as or better than previous pedometer-based interventions in which participants increased their step count by 2183 steps per day and decreased systolic BP by 3.8 mm Hg, diastolic BP by 0.3 mm Hg, and BMI by 0.38 kg/m^2^ from baseline on average [81]. Most of the meta-analyses have reported no statistically significant effect of walking on HDL cholesterol [81,85-87]. The improvement in biomedical outcomes in this study could be associated not only with the increase in physical activity but also with changes in diet.

Most aspects of self-regulation were enhanced both at P1a and P2a. It is possible that enhancement of self-regulation itself, rather than the mediating effect of self-efficacy, may have led to an increase in step count. Factors of the 12-item Physical Activity Self-Regulation scale, except social support, correspond with each function of DialBetes Step; for example, functions of positive feedback, action planning, and barrier identification and problem-solving were targeted for reinforcements, time management, and relapse prevention, respectively. Previous reviews revealed that interventions with a goal-setting component had large effects on physical activity [70,81,88]. Although it is difficult to identify the active components of such a complex intervention [17], our results including the subjective usefulness of each function suggest that goal setting [89], weekly feedback [90], and self-monitoring [31,89] were useful in increasing step count.

Despite the significant increase in steps per day in the short-term analysis, attenuation was observed at P2b. Even in weeks 21 to 22 before the long holidays, in which the increase was statistically significant, the median change from P0b was less than that during P1b. Previous studies often reported a trend toward a slight decrease in step count after the peak around the second or third month of interventions [66,91-94]. Self-efficacy is reported to be a mediator for the effects of interventions on physical activity [31], including future habituation [43]. Our participants’ inability to maintain the increase in step count may be attributed to the insufficient improvement in self-efficacy. Action planning is reported to be significantly associated with positive changes in self-efficacy [95,96]; however, our participants’ rating of action planning was relatively low. Moreover, interventions providing vicarious experience and feedback by comparison with others’ performance and the user’s past performance produced large effects on self-efficacy [30]. It will be necessary to improve the action planning function and add social functions in future versions of the system to enhance users’ self-efficacy.

The results of adverse event assessment suggest a need for some other improvement in the system. Despite no apparent adverse effects caused by using DialBetes Step, a participant experienced considerable pain in their lower limbs. Countermeasures against the worsening of body pain should be implemented, such as periodic pain assessment and advising against increase in physical activity when necessary. Another participant’s symptoms caused by insufficient sleep indicated that the system use was a burden on busy workers. The mean time spent in using the system in this study was not different from that in our previous study of the original DialBeticsLite [15]; participants used most of their time for measurement and recording; therefore, we should try shortening that time by using easy recording platforms and minimizing difficulties.

To the best of our knowledge, there are few SCT-based mobile apps designed to promote physical activity. We developed original goal-setting algorithms that intended to enhance users’ self-efficacy by automatically suggesting incremental goals tailored for each user. Our future exploration of the participants’ goal-setting processes might add new findings to the currently limited evidence of appropriate step goals [97].

### Limitations

This study has some limitations. First, participants in this study were not consistent with the initially defined theoretical target population of DialBetes Step. Despite an entry criterion of “not walking enough in general,” most participants (21/30, 70%) had already reached the national goal of steps per day [10] at P0b. Although their self-reported frequencies of self-regulation at P0a were lower than those of general workers across Japan [76], they may have been physically active because of their workplace and commuting environment where workers have many opportunities to walk (ie, department store in metropolitan Tokyo). Goal-related self-efficacy at P0a was also high; therefore, the changes might have been underestimated because of the ceiling effect. Although the post hoc subgroup analyses suggested that less-active participants were more likely to increase their steps per day for a short period, subsequent studies should evaluate the effects of the system in sedentary people.

Second, the results of outcome evaluation might be biased for various reasons. First, a few participants were lost to follow-up or those without data were excluded from analyses. As a previous review attributed the risk of bias partly to high levels of attrition [98], we should consider a possibility that the positive results were overestimated because the excluded participants were likely to have low adherence to the intervention. Nevertheless, we had a low level of attrition than previous interventions [98], which is a strength of this study. Second, measuring each outcome variable at a slightly different point in time made it difficult to interpret the results. For example, we had 2 time points (P0a or P0b) for baseline data collection; however, we asked participants not to change their behavior, and their steps per day did not change during P0b. We should note that the participants’ status would be different, even in the same period of outcome evaluation. Third, the intervention included not only mHealth system use but also face-to-face group sessions, which increased participants’ commitment to and utility of human resources in health care. This might limit the generalizability and scalability of the intervention.

Third, there might have been measurement errors in the accelerometer used in this study, which was reported to be less accurate in counting the number of steps than other frequently used pedometers and accelerometers in research settings [99]. The accuracy of the accelerometer had been examined only under controlled conditions using a treadmill at speeds between 70 and 120 m/min, not in uncontrolled environments [67]. The accelerometer might not have counted an irregular gait in daily activities precisely and might have counted hand movement as steps.

Finally, we could not assess the change in the intensity of walking. The intensity of physical activity is just as important as the amount [100,101]. Physical activity and clinical guidelines recommend moderate-intensity activity, such as moderate walking [6,102]. In the future, a function to record the parameters of walking intensity should be added for a more comprehensive assessment of physical activity.

### Conclusions

The intervention using DialBetes Step may be feasible and useful for increasing workers’ step count through self-regulation enhancement for a short period and, consequently, improving their BP and BMI. However, without significant change in self-efficacy, the increase in steps per day attenuated at the end of 24-week intervention. Future studies should improve the system with more self-efficacy–enhancing techniques and evaluate the effectiveness of the SCT-based new functions through a randomized controlled trial.

## Appendix

Multimedia Appendix 1 Outline of behavior change techniques used in the intervention.

Multimedia Appendix 2 Lists of actions for increasing step count, current and future barriers to walking, and possible solutions for each barrier provided by DialBetes Step.

Multimedia Appendix 3 Flowchart of deciding the frequency of blood glucose level measurements.

Multimedia Appendix 4 Detailed information about measuring instruments and scales used in the study.

Multimedia Appendix 5 Supplementary tables about the results of the study.

Multimedia Appendix 6 Distribution of changes in the mean steps per day from the baseline system use period.

## Abbreviations

BP: blood pressure
CVD: cardiovascular disease
ES-SMBPA-2D: evaluation scale for self-management behavior related to physical activity of type 2 diabetic patients
HDL: high-density lipoprotein
mHealth: mobile health
P0: baseline
P0a: before the intervention
P0b: baseline system use period
P1: around week 6
P1a: after 6 weeks of the intervention period
P1b: weeks 5 to 6
P2: the end of the intervention
P2a: after the intervention
P2b: weeks 23 to 24
SCT: Social Cognitive Theory
VFA: visceral fat area

## Data Availability

The data sets generated and analyzed during this study are not publicly available because we did not obtain consent from participants for data sharing. They will be available from the corresponding author upon reasonable request and after approval by the research ethics committee.
