# Supplementary material for: Preliminary Efficacy, Feasibility, and Perceived Usefulness of a Smartphone-Based Self-Management System With Personalized Goal Setting and Feedback to Increase Step Count Among Workers With High Blood Pressure: Before-and-After Study
Source: JMIR Cardio. 2023 Jul 21;7:e43940. doi: 10.2196/43940 (PMC10403795; doi:10.2196/43940)
Supplement: Multimedia Appendix 1 [file cardio_v7i1e43940_app1.docx]

Table. Outline of behavior change techniques used in the intervention.

| Time / period | Date | Intervention | | | |
| --- | --- | --- | --- | --- | --- |
|  |  | Description | Frequency | Function | Behavior change techniques^a^ |
| Educational group session  (2 hours) | The end of October 2018^b^ | Lecture on hypertension and its management (by the first author): definition, epidemiology, complications, the home measurement of BP,^c^ recommended diet (reducing sodium intake, eating vegetables and dietary fiber, limiting alcohol consumption), aerobic exercise (mainly moderate walking), weight management | Once (40 minutes) | Conventional intervention | 1.1. Goal setting (behavior) |
|  |  |  |  |  | 1.3. Goal setting (outcome) |
|  |  |  |  |  | 4.1. Instruction on how to perform a behavior |
|  |  |  |  |  | 5.1. Information about health consequences |
|  |  |  |  |  | 5.6. Information about emotional consequences |
|  |  | Lecture with a practice session on how to use DialBetes Step (by the first author with some supporting staff, such as a registered dietician) | Once (60 minutes) | Conventional intervention |  |
| Baseline system use period  (2 weeks) | November 1 to 14, 2018 | Measurement and recording of BP, body weight, and blood glucose levels | Daily | Self-monitoring (conventional intervention) | 2.4. Self-monitoring of outcomes of behavior |
|  |  |  |  |  | 2.7. Feedback on outcomes of behavior |
|  |  | Measurement and recording of daily step count and calories burned by activity | Daily | Self-monitoring (conventional intervention) | 2.2. Feedback on behavior |
|  |  |  |  |  | 2.3. Self-monitoring of behavior |
|  |  | Messages prompting users to continue measurement and recording (only after recording daily step count) | Daily | Self-monitoring | 2.3. Self-monitoring of behavior |
|  |  |  |  |  | 2.4. Self-monitoring of outcomes of behavior |
|  |  | Input of diet and exercise that is not recorded by the accelerometer | Daily | Self-monitoring (conventional intervention) | 2.2. Feedback on behavior |
|  |  |  |  |  | 2.3. Self-monitoring of behavior |
| Intervention period  (24 weeks)^d^ | November 20, 2018 to May 6, 2019 | Weekly feedback on step count, BP, body weight, and blood glucose levels | Weekly | Positive feedback | 2.2. Feedback on behavior |
|  |  |  |  |  | 2.7. Feedback on outcomes of behavior |
|  |  |  |  |  | 10.4. Social reward |
|  |  |  |  |  | 10.10. Reward (outcome) |
|  |  | Goal setting for daily steps | Weekly | Goal setting | 1.1. Goal setting (behavior) |
|  |  |  |  |  | 1.5. Review behavior goal |
|  |  |  |  |  | 8.7. Graded tasks |
|  |  | General information to increase step count (action list: options for the action plan) | Weekly^e^ | Information delivery | 3.2. Social support (practical)^f^ |
|  |  |  |  |  | 4.1. Instruction on how to perform a behavior |
|  |  | Action planning to achieve step goals | Weekly^e^ | Action planning | 1.4. Action planning |
|  |  | Identification of barriers to walking and possible solutions | Weekly^e^ | Barrier identification and problem-solving | 1.2. Problem solving |
|  |  |  |  |  | 3.2. Social support (practical)^f^ |
|  |  |  |  |  | 8.2. Behavior substitution^g^ |
|  |  | Self-rating of action plan achievement | Weekly | Action planning / barrier identification and problem-solving | 2.2. Feedback on behavior |
|  |  |  |  |  | 2.3. Self-monitoring of behavior |
|  |  |  |  |  | 10.4. Social reward |
|  |  | Evaluation messages on BP, body weight, and blood glucose levels | Daily | Conventional intervention | 2.7. Feedback on outcomes of behavior |
|  |  | Evaluation and general advice on diet | Daily | Conventional intervention | 1.6. Discrepancy between current behavior and goal |
|  |  |  |  |  | 2.2. Feedback on behavior |
|  |  |  |  |  | 4.1. Instruction on how to perform a behavior |
|  |  |  |  |  | 3.2. Social support (practical)^f^ |
|  |  |  |  |  | 5.1. Information about health consequences |
|  |  | Daily feedback on the user’s step goal achievement | Daily | Positive feedback | 1.6. Discrepancy between current behavior and goal |
|  |  |  |  |  | 2.2. Feedback on behavior |
|  |  |  |  |  | 10.4. Social reward |
|  |  |  |  |  | 10.9. Self-reward |
|  |  | Individualized information for safe physical activity | About once in 2 weeks | Information delivery | 4.1. Instruction on how to perform a behavior |
| From week 3 of the intervention period |  | Feedback on the user’s favorable physical activity behaviors (when the user meets their step goal) | 5 days a week | Positive feedback | 2.2. Feedback on behavior |
|  |  |  |  |  | 10.4. Social reward |
|  |  | Individualized advice to promote physical activity (when the user does not meet their step goal) | 5 days a week | Information delivery | 4.1. Instruction on how to perform a behavior |
|  |  |  |  |  | 3.2. Social support (practical)^f^ |
|  |  |  |  |  | 8.2. Behavior substitution^g^ |
|  |  |  |  |  | 12.1. Restructuring the physical environment^h^ |

^a^Defined in the Behavior Change Technique Taxonomy version 1 [49].

^b^Participants selected the day of attendance from 2 options. Because 3 participants could not attend the educational group session, they were provided a similar individual session and started using the system 1 to 5 weeks later.

^c^BP: blood pressure.

^d^Participants also used the same functions as the baseline system use period. Messages prompting users to continue measurement and recording were provided once a week for the initial 4 weeks, every 2 weeks for the next 8 weeks, then every 4 weeks, and when necessary.

^e^The system automatically chooses between action planning with general information to increase step count or barrier identification and problem-solving.

^f^The action list, a solution list, an item of diet advice, and an item of individualized advice to promote physical activity include strategy to arrange social support.

^g^A solution list and an item of individualized advice include strategy for behavior substitution.

^h^An item of individualized advice includes strategy to restructure the physical environment.
