# Supplementary material for: Preliminary Efficacy, Feasibility, and Perceived Usefulness of a Smartphone-Based Self-Management System With Personalized Goal Setting and Feedback to Increase Step Count Among Workers With High Blood Pressure: Before-and-After Study
Source: JMIR Cardio. 2023 Jul 21;7:e43940. doi: 10.2196/43940 (PMC10403795; doi:10.2196/43940)
Supplement: Multimedia Appendix 2 [file cardio_v7i1e43940_app2.docx]

Textbox S1. Action list for increasing step count provided by DialBetes Step.

| - Getting off the train or the bus early and walking the rest of the way - Moving as often as possible at the workplace or home - Visiting the restroom on the far side of the building - Walking after a meal - Walking on a lunch break - Walking on days off - Going out on days off - Lengthening time spent walking - Increasing the frequency of walking - Using exercise facilities (eg, a gymnasium) - Using a treadmill at home - Making time for walking with friends, family members, or co-workers |
| --- |

Table S1. Lists of current barriers to walking and possible solutions for each barrier provided by DialBetes Step.

| Current barriers | Solutions |
| --- | --- |
| I am tired. | Walking before feeling tired (eg, in the morning or the daytime) |
|  | Walking on days off without strain |
| Walking is boring. | Inviting friends, family members, or co-workers to join me for a walk |
|  | Engaging in leisure activities where I can walk (eg, shopping and museum tours) |
| I do not have enough time. | Avoid using transportation (including cars and elevators) for short travel and walking instead |
|  | Walking during small chores or errands |
| The weather is bad. | Walking inside (eg, underground streets, department stores, and exercise facilities) when the weather is bad |
|  | Using a treadmill at home when the weather is bad |
| I am anxious about hypoglycemia. | Measuring blood glucose levels before and after walking |
|  | Consulting a physician |
| I do not want my muscles to be sore. | Warming-up before and cooling-down after walking |
|  | Increasing walking time gradually, not rapidly |

Table S2. Lists of future barriers to walking and possible solutions for each barrier provided by DialBetes Step.

| Future barriers | Solutions |
| --- | --- |
| When I become tired of walking. | Thinking of pleasant walking courses for a change of scenery |
|  | Thinking of leisure activities where I can walk (eg, shopping and museum tours) |
| When I feel depressed. | Avoid stress by not working too hard |
|  | Making walking a habit in daily life |
| When I am busy and do not have enough time. | Making walking a habit in daily life |
|  | Thinking of small chores or errands involving walking even within my busy schedule |
| When the weather is bad. | Thinking of places and ways to walk when the weather is bad (eg, indoor facilities) |
|  | Preparing clothes and shoes suitable for walking in bad weather |
| When the season changes (to summer or winter and it gets hot or cold). | Preparing outfits appropriate to the season (eg, winter clothes and a water bottle) |
|  | Looking for places to walk comfortably even in hot or cold seasons |
| When I stop using DialBetes Step after participation in the study. | Preparing a tool to measure steps after the study ends (eg, a pedometer and my smartphone or mobile phone) |
|  | Thinking of ends and ways of continuing walking after the study |
|  | Learning ways to increase or maintain the number of steps while participating in the study |
