# Supplementary material for: Preliminary Efficacy, Feasibility, and Perceived Usefulness of a Smartphone-Based Self-Management System With Personalized Goal Setting and Feedback to Increase Step Count Among Workers With High Blood Pressure: Before-and-After Study
Source: JMIR Cardio. 2023 Jul 21;7:e43940. doi: 10.2196/43940 (PMC10403795; doi:10.2196/43940)
Supplement: Multimedia Appendix 3 [file cardio_v7i1e43940_app3.pptx]

## Slide 1
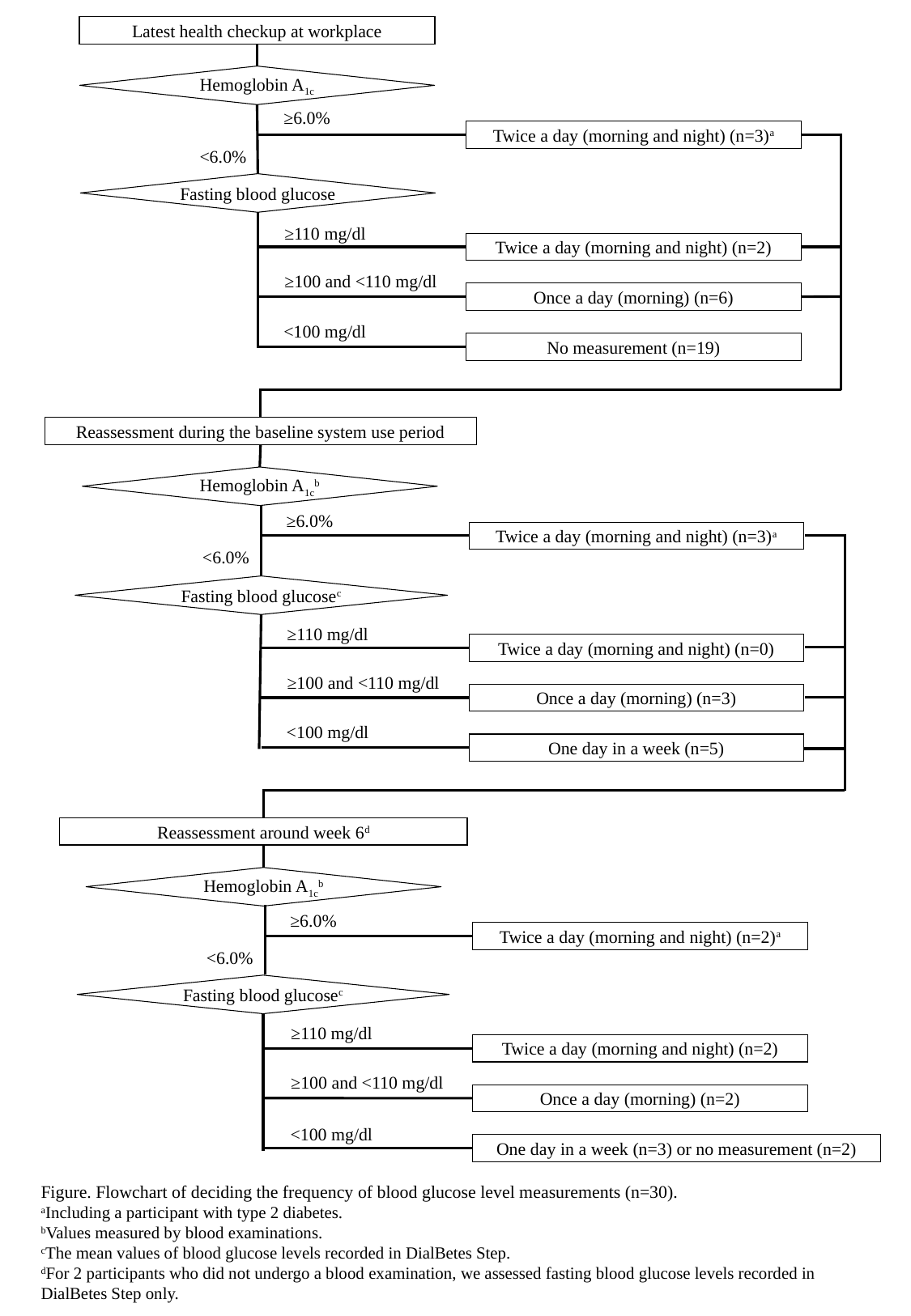

Latest health checkup at workplace
Hemoglobin A1c
≥6.0%
Twice a day (morning and night) (n=3)a
<6.0%
Fasting blood glucose
≥110 mg/dl
Twice a day (morning and night) (n=2)
≥100 and <110 mg/dl
Once a day (morning) (n=6)
<100 mg/dl
No measurement (n=19)
Reassessment during the baseline system use period
Hemoglobin A1cb
≥6.0%
Twice a day (morning and night) (n=3)a
<6.0%
Fasting blood glucosec
≥110 mg/dl
Twice a day (morning and night) (n=0)
≥100 and <110 mg/dl
Once a day (morning) (n=3)
<100 mg/dl
One day in a week (n=5)
Reassessment around week 6d
Hemoglobin A1cb
≥6.0%
Twice a day (morning and night) (n=2)a
<6.0%
Fasting blood glucosec
≥110 mg/dl
Twice a day (morning and night) (n=2)
≥100 and <110 mg/dl
Once a day (morning) (n=2)
<100 mg/dl
One day in a week (n=3) or no measurement (n=2)
Figure. Flowchart of deciding the frequency of blood glucose level measurements (n=30).
aIncluding a participant with type 2 diabetes.
bValues measured by blood examinations.
cThe mean values of blood glucose levels recorded in DialBetes Step.
dFor 2 participants who did not undergo a blood examination, we assessed fasting blood glucose levels recorded in DialBetes Step only.
