# Supplementary material for: Preliminary Efficacy, Feasibility, and Perceived Usefulness of a Smartphone-Based Self-Management System With Personalized Goal Setting and Feedback to Increase Step Count Among Workers With High Blood Pressure: Before-and-After Study
Source: JMIR Cardio. 2023 Jul 21;7:e43940. doi: 10.2196/43940 (PMC10403795; doi:10.2196/43940)
Supplement: Multimedia Appendix 4 [file cardio_v7i1e43940_app4.docx]

Table. Detailed information about measuring instruments and scales used in the study.

| Variable | Means of measurement | Name of the device / scale | How to measure / answer | Number of items | How to calculate the mean value / score | Accuracy / validity |
| --- | --- | --- | --- | --- | --- | --- |
| Steps per day | Accelerometer | Terumo;  MT-KT02DZ | By a triaxial acceleration sensor | N/A | Valid data for 3 days or more in each evaluation period was required for calculation. We excluded data from days on which the participant did not wear the accelerometer according to their self-report (ticks in paper-based recording sheets) or had daily step count <100 steps. | Accuracy was confirmed when it was worn in chest pockets or bags; there were no significant differences from the actual counted number of steps [67]. |
| Calories burned by activity per day | Accelerometer | Terumo;  MT-KT02DZ | From the intensity of walking measured by a triaxial acceleration sensor | N/A | Same as steps per day | Calculated values were highly correlated with the amount of energy consumption calculated from expired gas measurement in all wearing positions (*r*=0.84-0.90) such as chest and leg pockets and bags [67]. |
| Home blood pressure | Home sphygmomanometer | Omron;  HEM-7271T | Oscillometric method | N/A | When there was more than 1 measurement at one time, the mean value of the first 2 measurements within 15 minutes was regarded as the representative value for that occasion. Measurements before dinner were not counted and were excluded from the calculation of mean blood pressure at night. | Within ±3 mmHg |
| VFA^a^ | Medical device for visceral fat measurement | DUALSCAN (Omron Colin; HDS-2000) | Dual impedance method | N/A | N/A | Measurement values were highly correlated with VFA measured by radiographic computed tomography (*r*=0.89) [68,69], although DUALSCAN tended to underestimate VFA in overweight or obese people [69]. |
| Goal-related self-efficacy | Questionnaire | Newly developed scale by authors based on scales of self-efficacy for physical activity [71-73] | From 0% (cannot do at all) to 100% (absolutely can do) in multiples of 10% | 1 item with 4 levels of incremental step goals | The score was calculated as the average of the confidence estimates for 4 goals. | The baseline score was correlated with the mean steps per day in the baseline system use period among participants in this study (ρ=0.37, *P*=.045). |
| Self-efficacy in walking behavior | Questionnaire | Self-efficacy scale for walking behavior [74] | 5-point Likert scale of (1) “not at all” to (5) “completely” | 4 items | The total score is computed by summing up the scores of all items. | The scale showed good internal consistency and 1-month test-retest reliability and excellent construct validity among 30-49 years Japanese adults [74]. |
| Self-regulation for physical activity | Questionnaire | Japanese version of the 12-item Physical Activity Self-Regulation scale [75,76] | 5-point Likert scale of (1) “never” to (5) “very often” | 12 items (6 factors with 2 items each) | The total score and each factor score are computed by summing up the scores of the items. | The reliability and the construct validity of the scale were demonstrated among Japanese workers [76]. |
| Self-management behavior related to physical activity | Questionnaire | Evaluation scale for self-management behavior related to physical activity of type 2 diabetic patients [53] | 5-point Likert scale of (0) “never” to (4) “always” | 32 items (9 factors with 2 to 5 items each) | Each factor score is computed by summing up the scores of the items. | The factor validity was acceptable. All factors showed a strong internal consistency (except for a factor “creating situation”) and good reproducibility [53] |
| Body pain | Questionnaire | 25-question Geriatric Locomotive Function Scale [77] | 5-point Likert scale of (0) “no pain” to (4) “severe pain” | 4 items in the body pain cluster | The cluster score is computed by summing up the scores of the items. | The construct validity of the scale was confirmed and all items offered substantial test-retest reliability among Japanese elderly people [77]. |

^a^VFA: visceral fat area.
