# Supplementary material for: Preliminary Efficacy, Feasibility, and Perceived Usefulness of a Smartphone-Based Self-Management System With Personalized Goal Setting and Feedback to Increase Step Count Among Workers With High Blood Pressure: Before-and-After Study
Source: JMIR Cardio. 2023 Jul 21;7:e43940. doi: 10.2196/43940 (PMC10403795; doi:10.2196/43940)
Supplement: Multimedia Appendix 5 [file cardio_v7i1e43940_app5.docx]

Table S1. Percentages for the measurement and recording in DialBetes Step^a^ (n=30).

| Parameters | | Baseline system use period (for 14 days) | Intervention period (for 168 days) |
| --- | --- | --- | --- |
|  | | Percentages, median (IQR) | Percentages, median (IQR) |
| **Blood pressure** | |  |  |
|  | Morning | 92.9 (71.4-100) | 97.0 (87.5-98.8) |
|  | Night | 85.7 (50.0-100) | 86.9 (69.6-94.6) |
| Body weight | | 100 (85.7-100) | 97.3 (89.3-99.4) |
| Daily step count^b^ | | 96.4 (85.7-100) | 97.0 (88.7-100) |
| **Diet** | |  |  |
|  | Breakfast | 92.9 (71.4-100) | 97.3 (68.5-98.8) |
|  | Lunch | 92.9 (78.6-100) | 94.9 (79.2-99.4) |
|  | Dinner | 92.9 (64.3-100) | 94.9 (81.0-99.4) |

^a^Calculated by dividing the number of days data were measured and recorded by the number of days in each period.

^b^Days without data owing to recording failures, for which participants were not responsible, were excluded from the denominator.

Table S2. The number of contacts between participants and the study team.

| Categories | | Baseline system use period (for 14 days)^a^ | Intervention period (for 168 days) | Total |
| --- | --- | --- | --- | --- |
|  | | contacts, n (number of participants) | contacts, n (number of participants) | contacts, n (number of participants) |
| Email reminders to record accelerometer data (n=30) | | 13 (6) | 24 (11) | 37 (12) |
| **Problems with system use (n=34)** | | |  |  |
|  | Total | 21 (10) | 41 (22) | 62 (27) |
|  | Smartphones | 3 (3) | 0 (0) | 3 (3) |
|  | Measurement and recording of blood pressure | 4 (2) | 9 (8) | 13 (10) |
|  | Measurement and recording of body weight | 5 (3) | 7 (6) | 12 (9) |
|  | Measurement and recording of blood glucose (n=14) | 3 (1) | 7 (7) | 10 (7) |
|  | Measurement and recording of daily step count | 8 (7) | 23 (17) | 31 (22) |
|  | Diet recording | 4 (2) | 7 (4) | 11 (5) |

^a^Including the 5-day interval after the baseline system use period for the problems with system use (for 19 days).

Table S3. Subjective usefulness and user-friendliness of DialBetes Step.

| Functions | | 1 (good) | 2 | 3 | 4 (bad) |
| --- | --- | --- | --- | --- | --- |
|  | | n (%) | n (%) | n (%) | n (%) |
| **Usefulness (n=29)** | |  |  |  |  |
| Educational group session^a^ | | 11 (38) | 14 (48) | 2 (7) | 0 (0) |
| Overall system | | 16 (55) | 12 (41) | 1 (3) | 0 (0) |
| **Functions to increase step** **count** | |  |  |  |  |
|  | Measurement and recording of daily step count | 25 (86) | 4 (14) | 0 (0) | 0 (0) |
|  | Goal setting for daily steps | 15 (52) | 13 (45) | 1 (3) | 0 (0) |
|  | Messages after recording daily step count | 6 (21) | 21 (72) | 1 (3) | 1 (3) |
|  | Weekly feedback | 14 (48) | 14 (48) | 0 (0) | 1 (3) |
|  | Action planning | 9 (31) | 14 (48) | 5 (17) | 1 (3) |
|  | Barrier identification and problem-solving | 7 (24) | 15 (52) | 6 (21) | 1 (3) |
| **User-friendliness (n=28)^b^** | |  |  |  |  |
| Overall system | | 6 (21) | 19 (68) | 3 (11) | 0 (0) |
| **Functions to increase step count** | |  |  |  |  |
|  | Measurement and recording of daily step count | 15 (54) | 7 (25) | 5 (18) | 1 (4) |
|  | Goal setting for daily steps | 13 (46) | 12 (43) | 3 (11) | 0 (0) |
|  | Messages after recording daily step count | 6 (21) | 18 (64) | 4 (14) | 0 (0) |
|  | Weekly feedback^c^ | 13 (46) | 12 (43) | 2 (7) | 0 (0) |
|  | Action planning | 6 (21) | 16 (57) | 5 (18) | 1 (4) |
|  | Barrier identification and problem-solving | 5 (18) | 16 (57) | 5 (18) | 2 (7) |

^a^Total percentage is not 100% because of missing data (n=2).

^b^A participant who did not respond to user-friendliness items was excluded.

^c^Total percentage is not 100% because of missing data (n=1).

Table S4. Overall evaluation of DialBetes Step (n=29).^a^

| Statements | | Values |
| --- | --- | --- |
| I was able to use a smartphone with no problem (yes), n (%) | | 23 (79) |
| I was able to use a sphygmomanometer with no problem (yes), n (%) | | 29 (100) |
| I was able to use a weight and body composition scale with no problem (yes), n (%) | | 28 (97) |
| I was able to use a glucometer with no problem (yes) (n=11), n (%) | | 10 (91) |
| I was able to use an accelerometer with no problem (yes), n (%) | | 27 (93) |
| The interface of the system was easy to use (yes), n (%) | | 22 (76) |
| The user manual of DialBetes Step was easy to understand (yes), n (%) | | 26 (90) |
| I had difficulty incorporating using the system such as self-measurement and recording into daily practice (yes), n (%) | | 7 (24) |
| Technical problems were resolved within 24 hours (yes) (n=23), n (%) | | 18 (78) |
| **Measurement and checking my lifestyles gave me a sense of security, n (%)** | | |
|  | Absolutely yes | 14 (48) |
|  | Somewhat yes | 14 (48) |
|  | Somewhat no | 1 (3) |
|  | Absolutely no | 0 (0) |
| **I found the messages from the system motivating, n (%)** | |  |
|  | Absolutely yes | 4 (14) |
|  | Somewhat yes | 21 (72) |
|  | Somewhat no | 3 (10) |
|  | Absolutely no | 1 (3) |
| **I found the advice from the system useful, n (%)** | |  |
|  | Absolutely yes | 4 (14) |
|  | Somewhat yes | 20 (69) |
|  | Somewhat no | 4 (14) |
|  | Absolutely no | 1 (3) |
| **The system helped me to increase step count, n (%)** | |  |
|  | Absolutely yes | 23 (79) |
|  | Somewhat yes | 5 (17) |
|  | Somewhat no | 1 (3) |
|  | Absolutely no | 0 (0) |
| **Participation in the study helped me to improve lifestyle and self-management, n (%)** | | |
|  | Absolutely yes | 16 (55) |
|  | Somewhat yes | 11 (38) |
|  | Somewhat no | 1 (3) |
|  | Absolutely no | 1 (3) |
| Using the system took too much of my time (yes), n (%) | | 8 (28) |
| Using the system caused me some problems (yes), n (%) | | 5 (17) |
| Time spent using the system per day (minutes), mean (SD) [range] | | 16.5 (11.2) [3-60] |
| The system was worth the time I spent (yes), n (%) | | 26 (90) |
| **I want to continue using DialBetes Step, n (%)** | |  |
|  | Yes | 11 (38) |
|  | Somewhat yes | 10 (34) |
|  | Somewhat no | 8 (28) |
|  | No | 0 (0) |

^a^A participant who did not respond to the questionnaire was excluded.

Table S5. Subgroup analysis of the short-term change in the mean steps per day (n=29).^a^

| Subgroups | | n | P0b^b^, median (IQR) | Changes between P0b and weeks 5 to 6 | |
| --- | --- | --- | --- | --- | --- |
|  | |  |  | Median (95% CI) | *P* value^c^ |
| **Mean steps per day at P0b** | | | | |  |
|  | ≥10,000 | 15 | 11,519 (10,593-12,751) | +1958 (−544 to +3055) | .05 |
|  | <10,000 | 14 | 8486 (7889-8846) | +1451 (+111 to +3220) | .005 |

^a^A participant who was hospitalized during week 5 was excluded.

^b^P0b: baseline system use period.

^c^Analyzed using Wilcoxon signed rank test.

Table S6. Subgroup analysis of the long-term change in the mean steps per day (n=28).^a^

| Subgroups | | n | P0b^b^, median (IQR) | Changes between P0b and weeks 23 to 24 | |
| --- | --- | --- | --- | --- | --- |
|  | |  |  | Median (95% CI) | *P* value^c^ |
| **Mean steps per day at P0b** | | | | |  |
|  | ≥10,000 | 14 | 11,908 (10,954-12,751) | +859 (−662 to +2725) | .24 |
|  | <10,000 | 14 | 8486 (7889-8846) | +1484 (−1699 to +4453) | .14 |

^a^A participant without measured data in weeks 23 to 24 owing to accelerometer trouble was excluded.

^b^P0b: baseline system use period.

^c^Analyzed using Wilcoxon signed rank test.

Table S7. Changes in scores of the 25-question Geriatric Locomotive Function Scale (n=33).^a^

| Factors | P0a^b^, median (range) | Changes from P0a to P1a^c^ | | Changes from P0a to P2a^d^ (n=31) | |
| --- | --- | --- | --- | --- | --- |
|  |  | Median (95% CI) | *P* value^e^ | Median (95% CI) | *P* value^e^ |
| Total score^f^ | 5 (0-11) | 0 (−1 to 0) | .30 | 0 (−2 to 0) | .41 |
| Body pain^g^ | 1 (0-8) | 0 (−1 to 0) | .82 | 0 (−1 to +1) | .49 |

^a^A participant who did not respond to the questionnaire at P1a was excluded.

^b^P0a: before the intervention.

^c^P1a: after 6 weeks of the intervention period.

^d^P2a: after the intervention.

^e^Analyzed using Wilcoxon signed rank test.

^f^High score indicates bad locomotive functions (range 0-100).

^g^High score indicates great pain (range 0-20).

Table S8. Distribution of pain in lower limbs (n=33).^a^

| Factors | | P0a^b^, n (%) | P1a^c^, n (%) | P2a^d^ (n = 31), n (%) |
| --- | --- | --- | --- | --- |
| **Score of lower limb pain** | |  |  |  |
|  | 0 (no pain) | 22 (67) | 22 (67) | 22 (71) |
|  | 1 (mild pain) | 8 (24) | 8 (24) | 6 (19) |
|  | 2 (moderate pain) | 2 (6) | 1 (3) | 2 (6) |
|  | 3 (considerable pain) | 1 (3) | 2 (6) | 1 (3) |
|  | 4 (severe pain) | 0 (0) | 0 (0) | 0 (0) |

^a^A participant who did not respond to the questionnaire at P1a was excluded.

^b^P0a: before the intervention.

^c^P1a: after 6 weeks of the intervention period.

^d^P2a: after the intervention.
