# Supplementary figures and images for: Preliminary Efficacy, Feasibility, and Perceived Usefulness of a Smartphone-Based Self-Management System With Personalized Goal Setting and Feedback to Increase Step Count Among Workers With High Blood Pressure: Before-and-After Study
Source: JMIR Cardio. 2023 Jul 21;7:e43940. doi: 10.2196/43940 (PMC10403795; doi:10.2196/43940)

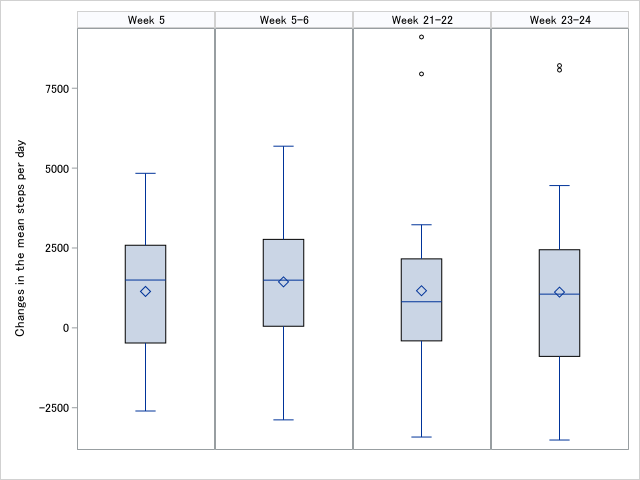

Supplement: Multimedia Appendix 6 [file cardio_v7i1e43940_app6.png]
